# DNA Oncogenic Virus-Induced Oxidative Stress, Genomic Damage, and Aberrant Epigenetic Alterations

**DOI:** 10.1155/2017/3179421

**Published:** 2017-06-27

**Authors:** Mankgopo Magdeline Kgatle, Catherine Wendy Spearman, Asgar Ali Kalla, Henry Norman Hairwadzi

**Affiliations:** ^1^Division of Hepatology, Department of Medicine, Faculty of Health Sciences, Groote Schuur Hospital, University of Cape Town, Cape Town, South Africa; ^2^Division of Rheumatology, Department of Medicine, Faculty of Health Sciences, Groote Schuur Hospital, Cape Town, South Africa

## Abstract

Approximately 20% of human cancers is attributable to DNA oncogenic viruses such as human papillomavirus (HPV), hepatitis B virus (HBV), and Epstein-Barr virus (EBV). Unrepaired DNA damage is the most common and overlapping feature of these DNA oncogenic viruses and a source of genomic instability and tumour development. Sustained DNA damage results from unceasing production of reactive oxygen species and activation of inflammasome cascades that trigger genomic changes and increased propensity of epigenetic alterations. Accumulation of epigenetic alterations may interfere with genome-wide cellular signalling machineries and promote malignant transformation leading to cancer development. Untangling and understanding the underlying mechanisms that promote these detrimental effects remain the major objectives for ongoing research and hope for effective virus-induced cancer therapy. Here, we review current literature with an emphasis on how DNA damage influences HPV, HVB, and EBV replication and epigenetic alterations that are associated with carcinogenesis.

## 1. Introduction

Carcinogenesis is a multistep process characterised by abnormal growth and proliferation of cells that result from repeated cycles of aberrant genetic and epigenetic modifications, which alter a substantial amount of human genes especially immunoregulatory, tumour suppressors, and oncogenes. It proceeds when the body's normal control machineries such as cell cycle arrest and programmed cell death are disrupted, leading to malignant transformation and subsequently tumour formation [1, 2]. Although all human cancers are noncommunicable diseases, approximately 20% of their global burden is attributable to DNA oncoviruses [2, 3]. Three well-studied DNA oncogenic viruses include human papillomavirus (HPV), hepatitis B virus (HBV), and Epstein-Barr virus (EBV). EBV, HBV, and HPV infections are associated with gastric, hepatocellular carcinomas, and cervical cancers, respectively [4]. Several mechanisms that underlie the ability of oncogenic viruses to transform normal cells to cancer cells have been described. Oncogenic viruses like HBV typically activate oxidative stress-mediated signalling mechanisms that influence an inflammatory cell migration leading to mutations and tissue damage [5, 6]. Some oncoviruses harbour tumorigenic or oncogenic materials that enable them to escape the host immune defence mechanisms and establish persistent infection. This ultimately predisposes to carcinogenesis by hijacking the host's checkpoint-controlling cell machineries [7, 8]. Host genomic instability and disrupted cellular machinery effects from genome-wide mutations and epigenetic alterations clearly seem accountable for the pernicious facet of oncovirus-induced carcinogenesis [9].

## 2. Oncogenic Virus-Induced Reactive Oxygen Species and DNA Damage

Chronic inflammatory responses triggered by persistent viral infection usually lead to unceasing production of reactive oxygen species (ROS). ROS are highly reactive oxygen-containing radicals expressed mainly by neutrophils and phagocytes as part of host defence mechanisms against pathogens [10]. ROS cooperates with reactive nitrogen species (RNS), and collectively, they form RONS [11]. RONS generates DNA damage and activation of DNA damage response (DDR) proteins that promote tissue injury at the site of inflammation. Under sustained viral-induced environmental stress, activated phagocytic cells may result in overexpression of RONS that alter the microenvironment and cause defective DNA repair and damage [12]. These effects are critically important and may lead to chromosomal alterations that promote genome instability, mutations, and epigenetic adaptations in cellular machinery systems that normally suppress tumorigenesis [3]. DNA viruses may encode oncogenic genes that are also capable of hijacking host cellular mechanisms to regulate cell survival and propagation. When these oncogenic genes overcome the ability of host cell machinery to control homeostasis, they trigger the tumour microenvironment associated with an elevated level of mutations that cause malignant transformation and ultimately cancer [8].

ROS regulates inflammasomes in response to oncogenic viral infections [10, 13, 14]. Inflammasomes are important component of the innate immune system that are recruited to a damaged site to facilitate tissue repair. They activate a key inflammatory mediator known as caspase-1 and promote inflammation in response to invading pathogens. Amongst several inflammasomes are interferon gamma-inducible protein 16 (IFI16), absent in melanoma 2 (AIM2), apoptosis-associated speck-like protein containing a CARD (ACS), NLR-family apoptosis inhibitory protein (NAIP), NOD-like receptor-family CARD domain containing protein 4 (NLRC4), NLR family pyrin domain containing 1 (NLRP1), and NLRP3 [15, 16]. Caspase-1 cooperates with ACS to induce inflammasomes by secreting proinflammatory cytokines pro-interleukin- (pro-IL-) 1*β* and pro-IL-18 into their active form IL-1*β* and IL-18 [15]. This influences cell survival by inducing pyroptosis through inactivation of IL-33, a transcriptional regulator of NF-*κ*B p65 subunit. AIM2 initiates caspase-1 activation and inflammasomes by binding to foreign cytoplasmic dsDNA and its adaptor ACS. Upregulated AIM2 significantly correlates with increased inflammation in chronic HBV infection, and this was found to induce renal damage in the presence of glomerulonephritis [17–19].

7,8-Dihydro-8-oxoguanine (8-oxoG) is a major form of oxidative DNA damage generated from ROS. Accumulation of 8-oxoG in the genome has been implicated in various inflammatory processes and human pathologies including viral infections [20]. 8-Oxoguanine glycosylase (OGG1), a DNA glycosylase enzyme, plays an important role in base excision repair (BER) pathway by repairing 8-oxoG-induced oxidised bases from dsDNA. OGG1 is downregulated under oxidative stress and leads to increased mutations of several immunoregulatory genes, oncogenes, and tumour suppressor genes [21]. It has also been shown that DNA double-strand break (DSB), another form of DNA damage, induces epigenetic abnormalities that are critical for carcinogenesis. Cancer epigenetics involves nonmutational alterations that regulate cellular identity and disrupt normal functions by abnormally switching “on” and “off” different genes. DNA methylation, histone modification, and noncoding mRNAs are special chemical marks of epigenetics that cooperate in determining whether a gene is accessible for reading, and they contribute to cancer initiation, progression, metastases, and chemoresistance. Epigenetic errors are driven by a spectrum of enzymes including DNA methyltransferases (DNMT1, DNMT3A, and DNMT3B), histone acetyltransferases (HATs), and histone deacetylases (HDACs). DNMTs are epigenetic writers that attach methyl groups to the nucleotide sequence whereas HDACs erase the acetyl groups from histones. Dimethylation of histone 3 lysine 9 (H3K9me2) and trimethylation of H3K9 (H3K9me3), H3K27me3, and H4K20me3 typically mark transcriptionally silent chromatin. These epigenetic mediators are important in normal development and cell differentiation. H3K27me3 and H4K20me3 are activated by enhancer of zeste homologue 2 (EZH2) and form part of polycomb repressive complex 2 (PRC2). PRC2 is one of the polycomb-group (PcG) proteins that regulate gene transcription by inducing epigenetics through EZH2, and it is associated with transcriptional repression of H3K27me2 and H3K27me3.

Intimate link between genome oxidative damage and epigenetics have been fairly described, and it suggests that oxidative DNA damage is a contributing factor in the induction of epigenetic alterations [22]. O'Hagan et al. [22] have shown that epigenetic drivers such as DNMT1, DNMT3b, EZH2, and SIRT1 are recruited at the site of DNA damage to establish and maintain silence in gene transcription. 5-hydroxymethylcytosine (5hmC), an oxidised product from 5-methylcytosine, was also shown to localise to the sites of endogenous DNA damage and promote repair, indicating its role in genomic integrity [23]. Zhang et al. [24] have recently demonstrated that knockdown of ten-eleven translocation (TET) 2 protein reverses aberrant oxidative stress-induced upregulation of DNMT1 and 5-hydroxymethylcytosine (5hmC) in cancer, suggesting a protective role of TET2 against DNA damage. Chromodomain helicase DNA binding protein 4 (CHD4) is a nucleosome remodelling and deacetylation (NuRD) enzyme. It is usually recruited to the DNA damaged sites to promote genome stability of DSBs and cell survival via interaction with rapid poly(ADP-ribosyl)ated proteins and ubiquitin ligase RING finger protein 8 [25–27]. CHd4 was also shown to regulate G1/S transition by regulating deacetylation of *p53* tumour suppressor gene that plays a central role in the DNA damage response. Induction of DSBs was observed in the promoter regions of genes such as *E-cadherin* and *RUNX3* that are aberrantly hypermethylated in several human malignancies [22, 28].

Following chronic infection and inflammation, overproduction of ROS induces DNA damage leading to the upregulation of DNMTs as part of the innate protective mechanism. DNMTs methylate circulating or integrated viral DNA resulting in the suppression of viral-encoded gene expression associated with reduced viral replication. Inappropriately, the same methylation system may also result in aberrant switching “on” and “off” important oncogenes and tumour suppressor genes that contribute to carcinogenesis [9, 29–31]. In this review, we cover epigenetic alterations and subsequent carcinogenesis induced by ROS-related DNA damage in HPV, HBV, and EBV infections.

## 3. Human Papillomavirus-Induced Carcinogenesis

Worldwide, cervical cancer is the second most common cancer affecting women. Approximately 500,000 women are diagnosed each year with cervical cancer and almost 300,000 die from the disease [32]. Cervical cancer begins as dysplasia, which is a slow developing abnormal cellular change that has the potential to become cancerous. Several cofactors such as parity, hormonal contraceptive diethylstilboestrol, long-term use of intrauterine device, early sexual relations and pregnancy before the age of 18, smoking, immunosuppression, family history, and chlamydia infection may increase the risk of cervical cancer. However, the most critical risk factor for cervical cancer is infection with sexually transmitted HPV. Nearly 100% cases of cervical cancers are caused by HPV, with about 630 million people infected worldwide [33, 34]. HPV infection targets epithelial basal cells in the skin, oropharyngeal and anogenital mucosae. More than a hundred types of HPV have been characterised, and they are classified into low- or high-risk HPV types [33]. HPV-1, HPV-6, and HPV-11 are low-risk types that cause foot and genital warts. HPV types are found mostly in benign and low grade squamous intraepithelial lesions [35]. High-risk HPV types 16, 18, 31, and 45 cause cervical cancer and are found in high-grade squamous intraepithelial lesions [35, 36]. The best-described types are HPV-16 that is associated with squamous cell carcinoma (SCC) and HPV-18 associated with adenocarcinoma [37].

HPV is a nonenveloped dsDNA virus of the genus *Alpha-Papillomavirus* in the Papillomaviridae family [36, 37]. HPV genome is approximately 8 kb in length. It contains eight ORFs with three functional coding regions including early expressed proteins E1-E7, late-expressed capsid proteins, and long control region (LCR) that lies between E1-E7 and capsid proteins [29, 38, 39]. L1 and L2 encode the major and minor capsid proteins that are important for packaging HPV genome into virion. They are composed of 72 pentameric (12 convex pentameric plus 60 planar hexameric) capsomeres that ultimately make up a total of 360 copies. These capsomeres are arranged on a skewed icosahedral surface lattice defined by the triangulation number of seven (*T* = 7) as first described in Casper-Klug's theory [40]. LCR is an 850 bp protein that contains a variety of elements that are important for regulating viral replication and gene transcription. Early genes regulate HPV DNA integration, replication, transcription, and transformation of epithelial cells. *E6* and *E7* are viral oncogenes that play a major role in immortality and malignant transformation of HPV-infected cells [41].

Mechanisms that facilitate HPV infection, replication, and progression to cervical cancer are well-documented. The transmission of HPV occurs mainly through direct skin-to-skin contacts during anal and vaginal intercourse [42]. The transformation zone of the cervix is the most common site of an HPV infection. The cervix is the lower end of uterus that extends into the vagina, and it becomes infected with HPV infection following sexual contact. The epithelial lining of the cervix consists of stratified squamous epithelial lining of the cervix, which is flat and more specialized or differentiated from basement membrane [43]. HPV infection begins when the virus penetrates the basal cells. Once the cells are infected, the viral capsid is shed and the circular HPV genome is shuttled into the nucleus of the cells. The basal cell layer houses the stem cells that replicate and undergo cell division and ultimately mature into squamous epithelial cells as they migrate to the upper epithelial layers [43–45]. Microabrasions or small tears sometimes occur in the lining of the cervical epithelium. This may allow potential HPV infectious viral particles to opportunistically invade the cervical epithelium and infect the basal layer cells. HPV DNA replicates and is maintained in the dividing basal cells as low copy episomal DNA. In rare instances, HPV DNA may integrate into host genome [43–45]. The dividing basal cells begin to differentiate, and viral genome amplification and synthesis of capsid L1 and L2 proteins occur in the upper layer of the epithelium. Papillomavirus infections are usually long-lived, and the dividing basal cells provide a reservoir of infected cells for the overlaying virus-producing tissue. This appreciation requires that the papillomavirus has an authentic and robust mechanism to replicate and retain the episomal genomes in the nuclei of the dividing cells. When the basal cells are programmed to differentiate, they activate the HPV virus to initiate stages of viral life cycle. These include reactivation of cell replication to produce multiple copies of viral DNA that, in turn, form the viral capsids, assembly, and finally the release of the virus [43–46].

HPV replication begins when the virus expresses its genes including *E6* and *E*7 [45]. The protein products formed from messenger RNA of these genes hinder the normal cell function activities and result in nonscheduled cell replication. These dysregulated cells are instructed to divide and produce multiple copy numbers of HPV viral DNA [47]. Consequently, nonscheduled replication causes an accumulation of damaged cells that interfere with the structural appearance of epithelial tissue that, once ordered and highly structured, now begins to look disorganised. The extension of these disorganisations through the upper layers and to the surface of the epithelium is used to classify the degree of the lesion. Aberrant cell growth in the lower one-third of the basal epithelium is categorised as cervical intraepithelial neoplasia 1 (CIN1) [47, 48]. When the damage extends up to two-thirds of the way from the bottom basal epithelium, it becomes CIN2. Ultimately, when the abnormally dividing cells occupy more than two thirds of the affected cell layer, it is referred to as CIN3. CIN3 is the immediate precursor to cervical cancer. Invasive cervical cancer ensues when the abnormal cells break through the basal membrane and infect the dermis. This results in viral persistence leading to accumulation of genomic instability and activation of various cellular genes that are critical in the transition of CIN2/3 precancerous lesions to invasive carcinoma [48, 49].

In normally dividing cells, two key cell cycle gene products prudently regulate the process. These include the *p53* and *retinoblastoma* (*pRB*). The *p53* is tumour suppressor and transcriptional factor that activates telomerase and protects the genome by preventing DNA degradation during replication and initiating DNA repair, cell cycle checkpoint, and apoptosis [45]. When bound to E2F, the *pRB* acts as a growth suppressor that regulates cell cycle and prevents damaged DNA replication. In high-risk HPV-type-infected cells, ORF E2 overexpresses early viral oncogenes *E6* and *E7*, which extend the cells' life span [50, 51]. These cells regain the capacity to proliferate a process known as immortalisation. During cell immortalisation caused by HPV, normal cellular mechanisms that protect cells from mutations are disabled and selective tumour suppressor genes are inactivated by subsequent epigenetic anomalies that trigger cervical cancer development [52].

### 3.1. Role of DNA Damage and Epigenetic Abnormalities in HPV-Induced Cervical Cancer

Infection with high-risk HPV types typically correlates with the progression of CIN2/3 precancerous lesions to invasive carcinoma. Integration of HPV DNA into the host DNA seems to be an important mechanism exploited by HPV oncoproteins to promote malignant transformation by evading immune responses and apoptosis. Recently, a study published in Nature Genetics provided insights in HPV integration-driven cervical tumorigenesis by conducting whole-genome sequencing and high-throughput viral integration detection [53]. This study revealed more than 3000 clustered genomic hotspots for HPV integration in cervical cancer. Viral integrants were detected into or close to various cellular genes that have been previously identified and novel ones including *DLG2*, *FHIT*, *HMGA2*, *KLF5*, *KLF12*, *LRP1B*, *LEPREL1*, *MYC*, and *SEMA3D* genes [53]. Alteration of gene transcription that promotes tumorigenesis in this study and others is evidence that HPV integration is an important aetiological event in the development of cervical cancer [53, 54]. HPV integration is facilitated by the DNA damage or DSBs induced by oxidative stress or disruption of viral E2 ORF, which results in the inactivation of E2 protein that negatively regulates the expression of *E6* and *E7* viral oncogenes [29]. This has recently been reviewed by Senapati et al. [55]. Inactivation of several DNA repair genes involved in various DNA repair pathways such as base excision repair (BER), nucleotide excision repair (NER), DNA mismatch repair (MMR), MMEJ, Fanconi anaemia (FA), ataxia-tengiectasia mutated (ATM), and the ATM and Rad3-related (ATR) may be implicated in HPV-induced cervical cancer. Microhomology-mediated end joining (MMEJ), the pathway for repairing DSBs in DNA, was observed between the HPV genome and integrants. This confirms the current hypothesis that induction of DNA DSBs by MMEJ indeed facilitates HPV integration and thus promotes tumorigenesis [56, 57].

**Figure 1:**
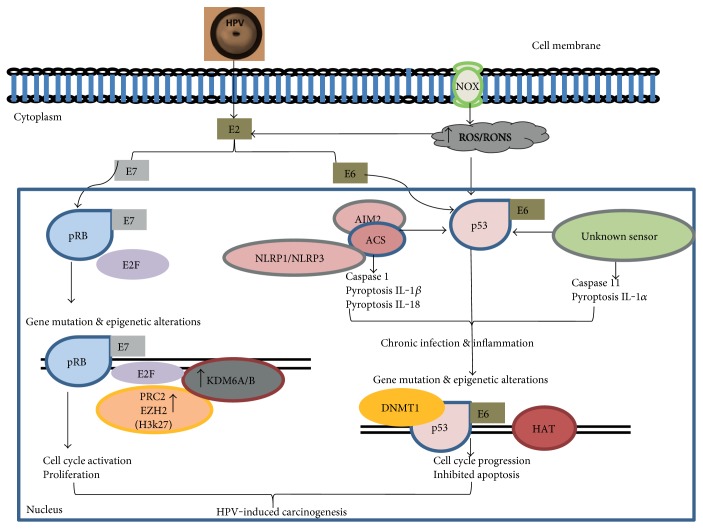
HPV-16 DNA integrates into the human genome at the E2 open reading frame. This in turn disrupts the transcriptional activities of E2 protein that negatively regulates the expression of E6 and E7 oncoproteins. Suppression of p53/pRB by normal upregulation of E6/E7 oncoproteins agitates multiple cellular signalling pathways that promote uncontrolled growth, proliferation, differentiation, and invasion of damaged cells and ultimately HPV-induced carcinogenesis. Unceasing expression of HPV-16 E6 oncogene correlates with increased accumulation of NOX-induced ROS/RONS DNA damage that activates inflammasome multimeric complexes that stimulate several caspases and upregulation of the proinflammatory cytokines IL-1*β*, IL-1*α*, and IL-18. Activation of inflammasomes causes genomic instability leading to gene mutations and epigenetic alterations that are critical for cell transformation and neoplastic progression.

Using colony formation assay, it was shown that upregulated HPV-16 promotes viral integration by activating the transcription of *E6* and *E7* oncogenes, which in turn increase the level of intracellular ROS/RONS leading to DNA damage in human keratinocytes as depicted in Figure 1 [7, 58, 59]. Elevated level of oxidative DNA damage as indicated 8-oxo-2′-deoxyguanosine coincides with increased HPV infection, viral-host integration, and dysplasia cervical lesion in cervical cells [60]. Nicotinamide adenine dinucleotide phosphate (NADPH) oxidases (NOXs) play a crucial role in the induction of E6/E7-mediated oxidation stress [61, 62]. NOX-mediated ROS is the major factor that mediates inflammasome activation in HPV infection [63]. Activation of HPV16-mediated AIM2 inflammasomes and upregulation of the proinflammatory cytokines IL-1*β*, IL-1*α*, and IL-18 were found to significantly increase cervical cancer progression in cervical lesions [14]. Interestingly, several single nucleotide polymorphisms (IL-1*β* rs1143643, IL-18 rs1834481, NLRP1 rs11651270, and NLRP3 rs10754558) are associated with protection against HPV infection, suggesting the important role of inflammasomes in oncovirus pathogenesis [13].

Apoptosis is an essential component and mode of programmed cell death that occurs normally in development as a defence mechanism to eliminate cells that are damaged by infections and disease [64]. During HPV infection, this process is hindered via disruption of apoptotic pathways that result in damaged cell proliferation leading to accumulation of mutations and abnormal gene alterations associated with tumour development. It is well known that p53 protein plays a key role in regulating genes involved in DNA repair system and activation of apoptotic pathways. Unfortunately, p53's protective role may be compromised during its interaction with HPV oncogenic protein/s during viral-host integration. E6 and E7 oncoproteins interact with p53 and pRB proteins, respectively. E6 binds to p53 protein resulting in its proteolytic degradation and inhibited apoptosis leading to the survival of HPV-infected cells with damaged DNA and ultimately genetic mutations or epigenetic anomalies that are associated with tumorigenesis (Figure 1). E7 usually binds and phosphorylates pRB protein, leading to activation of E2F transcription factor and unscheduled cell replication and division of episomal viral-infected cells. These, in turn, produce more viral particles and allow the viral DNA to integrate in the host DNA and trigger the introduction of mutations or epigenetic alterations.

Epigenetics marks an alteration in gene expression that is not associated with genome mutation. In HPV-16-related invasive cervical cancer, LCR and E2 binding sites at the URR are epigenetically regulated via hypermethylation [27, 65]. Methylation of these binding sites disrupts the function of E2 protein resulting in the upregulation of *E6* and *E7* oncogenes [65, 66]. Altered expression of *E6* and *E7* oncogenes via hypermethylation may serve as host defence mechanism to reduce or control viral replication in case multiple copies of HPV DNA are integrated into the host DNA [67, 68]. DNA methylation may also serve as methylation-related barricade device intended to disguise the virus from immune attack [30, 69]. Three important host epigenetic machinery methyltransferases DNMT1, DNMT3A, and DNMT3B have been implicated in HPV-16 activities. For instance, HPV-16 E6 oncoprotein promotes overexpression of DNMT1 by silencing the expression of *p53* tumour suppressor gene [70]. On the other hand, upregulation of DNMT3A and DNMT3B was activated by HPV-16 E7 oncoprotein [71, 72]. Upregulation of DNA methyltransferases by HPV-16 oncoproteins leads to methylation of both host and viral DNA. It has been shown that methylation of HPV and host DNA is more likely to be integration dependent. This was demonstrated by SCC samples with integrated HPV genome, which exhibited high heterogeneity of methylation levels (30–75%) especially in the CpG island promoter regions that are typically prone to methylation [68]. Reduced level of methylation was observed in the SCC samples with a single copy of integrated HPV-16 DNA as compared to multiple integrated HPV DNA copies that exhibited significantly increased methylation levels [73]. These findings suggest strong correlation between HPV integration and methylation levels and that these two parameters play an important role in HPV-related carcinogenesis. Epigenetic changes in HPV-16 were also found to occur through the interaction of HPV oncogenes with HAT [59, 74]. HPV-16 ORF E6 protein influences interaction of p53 with DNMT1 and pCBPAF. This complex disrupts p53 activities and benefits the virus by enhancing viral replication leading to cell cycle progression and the survival of HPV-infected cells with unrepaired damaged DNA [74]. Like in many human cancers, silencing of genes by promoter hypermethylation is also a common feature in HPV-related cervical cancer. Hypermethylation of genes such as *CDH1*, *DAPK*, *TIMP-3*, *p16(ink4a)*, *FHT1*, *RASSF1A*, *KATNAL2*, and *COL25AI* correlates with progression to HPV-related cervical cancer [75–77]. HPV-16 and HPV-18 E6 proteins also bind and inactivate apoptotic-related protein, human telomerase reverse transcriptase (*hTERT*), through proteasomal degradation by ubiquitin ligase E6 associated protein (E6-AP) [52, 78]. *hTERT* gene is hypomethylated in cervical cancer, and this correlates with poor prognosis [79]. *RASSF1A* plays an important role in the apoptotic signalling and DNA repair pathways. Transcriptional silence in *RASSF1A* gene via HPV-induced hypermethylation has been observed in cervical cancer, suggesting an important link between DNA damage and methylation [80].

HPV E7 targets E2F6, an important component of PRC2. In cervical cancer cells, overexpression of EZH2 correlates with cell growth, proliferation, and cancer progression suggesting poor patient survival [81]. It has recently been demonstrated that disruption in EZH2 expression reverses the chemotherapy drug resistance in cervical cancer cells partly by increasing the expression of Dicer [82]. Dicer is an enzyme that cleaves dsRNA into short interfering RNAs and microRNAs (miRNAs). Epigenetic reprogramming of host epithelial cells through increased expression of KDM6A and KDM6B (jumonji domain containing 3) histone demethylase by HPV E7 oncogene has also been reported, and this was associated with dramatic reduction in trimethylation of H3K27. Silence in the expression of EZH2 reversed the effects of KDM6A and KDM6B, confirming the important role of EZH2 during HPV infection and cervical cancer [83]. HOX transcript antisense RNA (HOTAIR) is a long intergenic noncoding RNA that targets PRC2 and LSD1. Interaction of HOTAIR with HPV-16 E7 oncogene was found to alter the expression and function of HOTAIR in E7-transfected cervical cancer cell lines [84]. Overexpression of HOTAIR in cervical cancer is associated with poor patient prognosis, suggesting a potential target for diagnosis and gene therapy [85].

Though the role of miRNAs (miRs) in HPV-related cervical cancer remains unclear, aberrant alteration of a group of miRNAs has been observed. However, the existing data is inconsistent due to heterogeneous expression levels being reported by various studies [86]. A group of the miRs detected in HPV-related cervical cancer includes *miR-7*, *miR-10a*, *miR-13*, *miR-17-5p*, *miR-19a*, *miR-19b*, *miR-20*, *miR-21*, *miR-133b*, *miR-138*, and *miR-196a*. These *miRs* have been found to downregulate several genes (*PCD4*, *CCL20*, *CHL1*, *CUL5*, *TNK52*, *XIAP*, *TP53INP*, and *P3IK*), most of which usually undergo DNA methylation in cervical cancer and regulate cell growth, proliferation, and apoptosis, mechanisms that are critical in HPV-related tumorigenesis.

## 4. Hepatitis B Virus-Related Hepatocarcinogenesis

Hepatoepigenetics triggered by chronic infection and inflammation in HBV has been recently described in the review authored by us and published in Biomedical Central International [87]. Chronic HBV (CHB) is the silent killer and the leading cause of liver cancer worldwide. Currently, more than 400 million people are CHB infected globally. CHB is associated with long-term inflammatory changes that damage the host DNA through the release of ROS and Kupffer cells activation via NF-*κ*B and AP1 [5, 6]. The Kupffer cells activate hepatic stellate cells that produce extracellular matrix proteins and cytokines [88–91]. Repeating cycles of this activation and inflammation causes hepatocyte injury leading to cirrhosis characterised by regenerative nodules and irreversible fibrosis that may eventually lead to HCC. Genomic instability introduced by HBV-induced DNA damage, viral integration into the host DNA following unrepaired DNA damage, and epigenetic alterations have been frequently reported in several studies as important mechanisms driving HBV-induced hepatocarcinogenesis.

### 4.1. HBV-Induced DNA Damage, Viral Integration, and Hepatoepigenetics

Activation of inflammasomes and its effects in HBV pathogenesis and pathophysiology has been briefly explored in several studies. Hepatocytes abundantly express high levels of IFI16 and AIM2 caspase-1 that is activated by inflammasomes to process the pro-IL-1*β* and pro-IL-18 into their mature active form [92, 93]. Increased expression level of IFI16 was found to be associated with p53 activation and decreased HBV viral suppression, suggesting a potential protective mechanism against HBV infection [94]. Upregulation of AIM2 strongly correlates with HBV load and enhanced inflammation in CHBV patients [17, 18, 95]. Similar AIM2 effects were observed in patients with HBV-induced glomerulonephritis, suggesting an important central role of AIM2 in contributing to inflammatory damage and severe disease progression [19]. Downregulation of caspase 1 and ACS was reported in the peripheral blood mononuclear cells of CHBV patients compared to their normal counterparts, suggesting a mechanism in which activation of pro-IL-1*β* and pro-IL-18 occurs in subsequent to HBV infection [96]. Lipopolysaccharide- (LPS-) induced NLRP3 inflammasome activation and IL-1*β* induction through obstruction of ROS generation and NF-*κ*B pathway was found to be inhibited during CHBV infection. This correlates with suppression of innate immune responses and HBV-induced immune tolerance, suggesting an underlying mechanism for HBV evasion [97].

**Figure 2:**
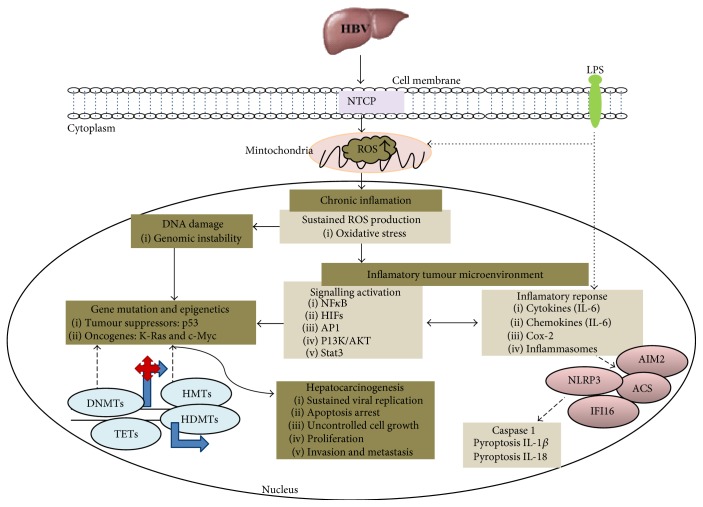
Chronic infection and inflammation occurs following repetitive cycles of HBV integration in the host genome. This results in overproduction of ROS production that generates DNA lesions, which promotes mutations and genome instability. Consequently, this activates a pool of inflammasomes (AIM2, ACS, NLRP3, and IFI16) and hepatoepigenetics via epigenetic regulators (DNMTs, TETs, HMTs, and HDMTs) that trigger aberrant transcriptional activities of hepatitis B x (HBx) protein and altered gene transcription leading to hepatocarcinogenesis.

Subsequent to ROS-induced DNA damage, HBV DNA integrates into host DNA introducing insertional mutagenesis, aberrant transcriptional activities of hepatitis B x (HBx) protein, and epigenetic alterations that lead to HCC progression (Figure 2). Nearly 90% of HBV-related HCC cases show evidence of HBV integration into the host genome [98]. HBV DNA integrates into the human genome soon after the repair and conversion of HBV DNA to cccDNA [9, 99–101]. Although the role of HBV integration remains controversial, several studies show that it is responsible for the “*cis*” and “*trans*” effects observed in host and viral genome, respectively. The “*cis*” effects incorporate insertional mutagenesis that are mediated by integrated virus and alter the normal functions of wide variety of host cellular genes that are critical in tumorigenesis. The “*trans*” effects target and alter the expression of viral genes with the aim of reducing viral replication [101]. The “*cis*” and “*trans*” effects of host and viral DNA are mediated by the transcriptional activities of HBx, which is also a potent epigenetic modifying regulator [101]. HBx protein is located in the nucleus and cytoplasm of HBV-infected cells in which it alters the transcription and functions of cellular genes by interacting with nuclear transcription regulators (e.g., CREB-binding protein/p300) or activating cytoplasmic signal transduction pathways [102]. HBx protein also interacts with cell cycle and DNA repair regulator known as UV-damaged DNA binding protein 1 (DDB1). This interaction stimulates viral replication by inducing chromosome segregation defects and genetic instability in regenerating hepatocytes that promote HCC development [103]. DDB1 acts as an adaptor for Culin4 (CUL4) as part of an E3 ubiquitin ligase complex. HBx hijacks CUL4-DDB1 complex to promote the proteasomal degradation of the structural maintenance of chromosome 5/6 (Smc5/6) complex. This increases viral replication and predisposes the infected hepatocytes to genetic or epigenetic anomalies under the condition of DNA damage [104–106]. It has been demonstrated that HBV genome integrates within the coding sequence or close to an array of key regulatory cellular genes that can deregulate proto-oncogenes and tumour suppressor genes. Activation or inactivation of such genes by enhanced ROS induction promotes genomic chromosomal instability, deletions, and chromosomal translocations in the host cells by altering various cellular signalling pathways [12, 106, 107]. Consequently, this will trigger genetic mutations and epigenetic alterations with a malignant phenotype (Figure 2).

In 47% of HBV-related HCC patients, HBV integration was detected in the *hTERT* locus, and this was closely associated with *hTERT* promoter mutations [108]. This suggests a tight link between viral integrants and critical regulators of DNA damage response pathway such as *hTERT*. Several studies in woodchucks and California ground squirrels (*Spermophilus beecheyi*) show that HBV genome integrates close to *ras* and *myc* family oncogenes such as *c-myc*, *N-myc1*, and *N-myc2*, which regulate cell proliferation and transformation [109]. Using a novel high-throughput viral integration method, HBV was found to integrate into 8q24 locus located *c-Myc* and *plasmocytoma variant translocation 1* (*PVT1*). HBV integration in these sites was observed in 12.4% of tissue samples with early-onset HBV-related HCC as compared to those with late-onset disease at a proportion of 1.4%. HBV integration in *c-Myc* and *PVT1* promoters was associated with aberrant overexpression of *c-Myc*, *PVT1*, and *miR-1204*, suggesting the importance of these integral sites in the early-onset hepatocarcinogenesis [110].

The *v-erb-A*, *cyclin A*, *RARβ*, *major histocompatibility complex I like leukocyte* (*Mill*), *platelet-derived growth factor receptor* (*PDGFR*), steroid receptor and calcium signalling-related genes are also common sites for HBV integration. These genes are important in cellular signalling pathways that control DNA damage, oxidation stress, and cell growth, and their alteration is associated with the development and progression of cancer [111, 112].

Methylation of HBV integrants tends to overlap methylation at flanking human genomic sequences. This was observed in PLC/PRF/5 and HepG2.2.15 cells which demonstrated that HBV genome becomes significantly methylated when integrated into highly methylated host genome region. HBV genome remains unmethylated when integrated into unmethylated host genomic regions such as promoters, and this may be associated with enhanced disease progression [113]. This observation is reminiscent of one of the most preferential targets of HBV DNA integration and potent regulator of DNA damage response pathway, *hTERT* gene. Transcriptional regulation of *hTERT* gene via methylation in HPV or HBV-related carcinomas as compared to normal controls has been observed [114, 115]. Although more evidence is still needed, the above observations suggest that there is a strong link between viral integration and epigenetic alterations and that genes involved in DNA damage repair machinery are also implicated. HBV-infected hepatocytes increase the expression of DNMTs as a host defence mechanism to reduce viral replication [9]. Long-term upregulation of DNMTs may eventually be implicated in the transcriptional activities of HBx and inappropriately methylate important host immunoregulatory or tumour suppressor genes that are critical in HBV-induced hepatocarcinogenesis [9].

HBx protein exerts its oncogenic ability by activating DNMT1, DNMT3, TETs, HMTs, HDMTs, and miRNAs resulting in the induction of epigenetic alterations of a spectrum of tumour suppressor gene promoters and miRNAs leading to HBV-induced HCC [87, 110, 116–121]. Loss of gene expression and perturbed cellular signalling pathways such DNA repair, transcription, cell growth, proliferation, and apoptosis that lead to malignant transformation, tumour initiation, progression, and metastases are common phenotypes associated with HBx-induced hypermethylation [87, 110, 116–121]. Amongst genes frequently implicated in HBV-induced epigenetic suppression are *p14* (*ARF*), *p15* (*INK4B*), *p16* (*INK4A*), and *pRB* [87, 119, 120].

Epigenomic studies show that DNA methylation and histone modification together promote hepatocarcinogenesis by regulating gene expression. In the case of HBV-related HCC, accumulation of HBV-induced ROS influences hepatocarcinogenesis by establishing epigenetic silence in the *suppressor of cytokine signalling 3* (*SOCS3*) promoter through upregulation of Snail expression and activation of DNMT1 and HDAC1 in HBV-related HCC. This coincides with sustained activation of IL-6/STAT3/SOCS3 signalling pathway that usually activates an inflammatory response and plays a regulatory role in hepatocarcinogenesis [122]. Recent study has shown that silence in the transcription of *SOCS3* gene may result from its genetic variants and hypermethylation, and this may correlate with disease susceptibility, poor prognosis, and development of HBV-related HCC [123]. HBV-induced methylation of single CpG in the 5′-untranslated region of *TRIM22* gene was found to be associated with suppression of gene transcription. *TRIM22* is an interferon-stimulating gene that plays an important role in the early host immunity against invading infections. Hypermethylation of *TRIM22* gene in HBV infection suggests an important mechanism by which HBV escapes the host innate immunity [124].

## 5. Epstein-Barr Virus-Induced Carcinogenesis

EBV is a dsDNA gammaherpesvirus of the *Lymphocryptovirus* genus, and it is associated with Burkitt's lymphomas (BL), Hodgkin's lymphomas (HL), nasopharyngeal (NC) and gastric carcinomas [125–127]. EBV infection occurs mostly during childhood and remains asymptomatic due to immune-related activities. Nearly 90% of the adult population worldwide is currently EBV infected, and the most common route of infection is through intimate contact with saliva from an EBV-infected person. EBV targets complement receptor type 21 (CD21/CR21) and human leukocyte antigen (HLA) class II coreceptor that are expressed by nasopharyngeal B-cells and epithelial cells, respectively. The EBV-targeted receptors establish the primary infection through interactions with envelope glycoproteins gp350 and gp42, which trigger fusion and internalization of the virus membrane with the cell membrane [31, 128, 129].

**Figure 3:**
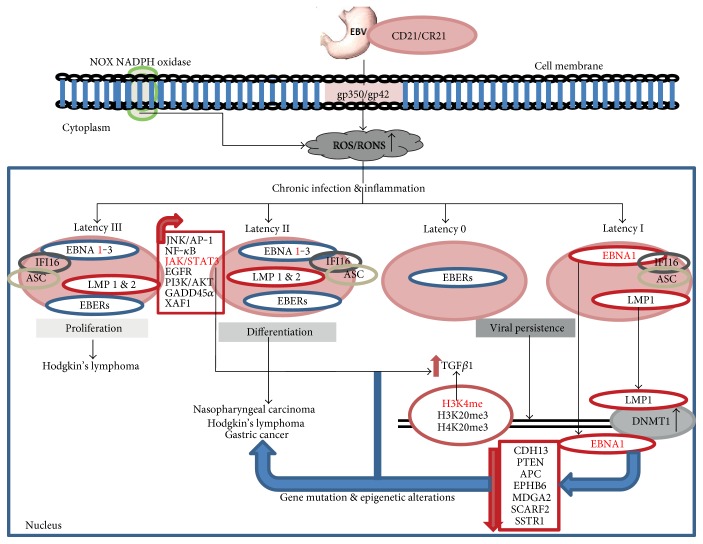
EBV-infected cells elicit reactive oxygen species- (ROS-) induced DNA damage via activation of NADH oxidase (NOX) family NADPH oxidase. This leads to persistent infection and inflammation via activation of inflammasome IFI16 and ASC that trigger posttranscriptional modifications of viral and host genes that are critical for promoting B-cell immortalisation, malignant transformation, and subsequently EBV-related tumorigenesis.

Upon viral infection, the EBV genome enters into the nucleus and circularises into an episomal form to establish persistent latent infection, an important step in initiating EBV-related tumorigenesis [31, 130]. During latent infection, various EBV genes including EBV nuclear antigens (*EBNA 1*, *2*, *3A*, *3B*, *3C*, and *LP*), latent membrane proteins (LMP 1, 2A, and 2B) and two EBV-encoded small RNAs (*EBER 1*, *2*, and transcripts from *BamHI A* region) are expressed [131–133]. The production of these genes follows the three different latency patterns (I, II, and III) important for cell survival, immortalisation, and proliferation (Figure 3). EBNA2 and LP are expressed soon during latent infection and synergistically disrupt several host cellular systems that abnormally control cell cycle and production of growth factors, leading to EBV-associated tumorigenesis [126]. LMP1 and EBNA 3A–C are intermittently expressed, and this usually correlates with induction of uncontrolled cell growth and proliferation while suppressing programmed cell death [132, 134]. EBNA3C regulates important host cellular processes such as cell growth, proliferation, and apoptosis during EBV latent infection, and this usually correlates with DNA damage. LMP1 and LMP2 are well-described oncogenic EBV proteins that promote malignant transformation of B-cells and epithelial cells. These proteins activate various cellular signalling pathways such as c-Jun N-terminal kinase and activator protein 1 (JNK/AP-1), nuclear factor of kappa-light-chain enhancer of activated B-cells (NF-*κ*B), Janus kinase/signal transducers and activators of transcription 3 (JAK/STAT3), and phosphatidylinositol 3-kinase (PI3K)/AKT [135].

### 5.1. The Role of EBV-Induced ROS, DNA Damage, and Epigenetic Alterations in Gastric Cancer

Although epigenetic alterations have also been observed in HL, BL, and NC carcinomas, the underlying mechanisms by which EBV introduces these alterations remain unknown [120, 136, 137]. Therefore, in this review, we will focus only in the effects of DNA damage and epigenetic alterations associated with EBV-related gastric cancer. Unlike HPV and HBV, EBV genome is not integrated into the host genome but maintained as an episome inside latently infected cells [33, 35]. EBV latency genes play an important role in gastric cancer by independently promoting genomic instability that triggers DNA damage by abnormally regulating cell cycle machinery and DNA repair of infected cells. Exposure of EBV-infected cells correlates with increased DNA damage induced by ROS-induced NOX and NADPH oxidase [138]. Several studies demonstrated that oxidative stress facilitates EBV-induced B-cell transformation through posttranscriptional regulation of viral (*LMP1*) and host (*STAT3*) genes, which are critical for promoting B-cell immortalisation, malignant transformation, and tumorigenesis [132, 138, 139]. IFI16 inflammasome with adaptor ASC protein was shown to be activated upon sensing of latent EBV infection in all types of latency, and this leads to induction of IL-1*β*, IL-18, and IL-33 maturation [140].

Vos et al. [141] in 1989 have shown that DNA damage promotes human cell transformation by integrative EBV-derived plasmid. In EBV infection, DNA damage signal transduction pathways such as ATM and caspase are activated leading to productive viral genome amplification and enhanced EBV-infected primary B-cell transformation efficiency [142, 143]. Although EBV is not integrated into host genome, its materials such as EBNA1 and BZLF1 seem to interact with various cellular promoters and induce epigenetic alterations. Epigenetic regulation of viral and host cellular growth-promoting factors such as EBNA1, BZLF1, STAT3, XIAP-associated factor 1 (XAF1), and DNA inducible factor 45 alpha (GADD45*α*) induces oxidative stress and expedites EBV-induced B-cell transformation [132, 138, 139, 144]. EBV-induced elevated level of ROS promotes excessive production of receptor activator of NF-*κ*B ligand (RANKL) that interacts with RANK receptor in the periapical area leading to bone resorption and apical periodontitis [145]. A significant ROS-induced reactivation of latent EBV infection was observed in Raj cells exposed to a crystalline organophosphate insecticide [146]. This was supported by an observation of increased expression level of BZLF1, an EBV immediate-early viral transcriptional activating protein that facilitates the switching between latent and lytic EBV life cycles [146–149]. Feng et al. [133] have shown that EBV BZLF1 protein binds to and activates DDR proteins. The same protein also induces oxidative stress-mediated DNA damage by interacting with p53 protein and impedes its activities via suppression cellular TATA binding proteins. *p53* is a long-standing tumour suppressor gene that maintains genome stability by providing protection from alteration-induced carcinogenesis. It was previously shown that inflammasome ASC serves an adaptor molecule that activates pyroptosis through p53-mediated regulation of BAX mitochondrial pathways [150]. By targeting the large tegument deneddylase protein to the nucleus, caspase-1 was shown to promote the accumulation of cullin-RING ligase that drives EBV replication [134]. Increased expression of mediated nuclear *IFI16* and *breast cancer 1* (*BRCA1*) genes was observed in EBV infection, and this coincides with enhanced activity of inflammasomes [18, 151, 152].

DNMTs, histone modifiers, chromatin remodellers, and polycomb group proteins are well-cited epigenetic regulators implicated in latent EBV replication. EBV latent *LMP1* induces hypermethylation of promoter regions *E-cadherin 13* (*CDH13*) and *docking protein 1* through activation of DNMT1 in EBV-related gastric cancer, leading to suppression of gene expression. *E-cadherin* gene expression was restored through treatment with 5-aza-20-deoxycytidine, a potent DNMT inhibitor, which led to reduction in rampant cellular growth and proliferation [153]. The activation of *LMP2* oncogene enhances upregulation of DNMT3B via EBV-induced hypermethylation at p*hosphatase and tensin homolog* (*PTEN*) CpG island promoter region [135]. *PTEN* has frequently been reported in several human malignancies as a tumour suppressor gene, which negatively regulates PI3K/AKT cellular signalling pathways. Methylation of *PTEN* promoter region in EBV-induced gastric tumorigenesis correlates with suppression of *PTEN* gene expression. Recently, it was revealed that increase in cellular TET enhances 5-hydroxymethylation by activating conversion of 5-methylated cytosine to 5-hydroxymethylcytosine, leading to dysregulation in lytic EBV viral reactivation [154]. TET enzymes are often deactivated in EBV-induced nasopharyngeal cancer. However, the mechanisms underlying this deactivation remain elusive.

The *BamHI W* promoter of EBV is susceptible to DNA methylation, and this alters the expression of EBNA1 protein. EBNA1 has been hypothesised to tether EPV episomes to host mitotic chromosomes and chromatin. High-throughput technology-based genome-wide studies demonstrated that EBNA1 interacts with HDACs. EBV-induced gastric adenocarcinoma is associated with promoter methylation of *p14, p16*, and *adenomatous polyposis coli* (*APC*) genes [155]. Other EBV-driven aberrant CpG island promoter hypermethylation in human gastric cancer include *EPH receptor B6* (*EPHB6*), *MAM domain containing glycosylphosphatidylinositol anchor 2* (*MDGA2*), *interleukin-15 receptor alpha* (*IL15RA*), *scavenger receptor class F member 2* (*SCARF2*), *somatostatin receptor 1* (*SSTR1*), and *Rec8 homolog* (*REC8*) (Figure 3) [156].

Trimethylation of H3K9 (H3K9me3), H3K27me3, H4K20me3, and H3K9me2 histone modifications play an important role during EBV latency and are associated with silent chromatin. Raji cell line models expressing EBV latent infection showed that H3K4me, H3K27me3, and H4K20me3 repress BZLF1 transcription, leading to reactivation of EBV [157]. This suggests a mechanism underlying the switch from latency to reactivation EBV. EZH2 is normally expressed in actively dividing cells and plays a critical role in cancer initiation, progression, and metastasis. The catalytic activity of EZH2 requires other PRC2 components including embryonic ectoderm development (EED), suppressor of zeste 12 (SUZ12), retinoblastoma-binding protein 4 (RBBP4), adipocyte enhancer binding protein 2 (AEBP2), and JARID2. EZH2 has a conserved feature of histone lysine methyltransferase C-terminal SET-domain whose function is to add a methyl group to lysine side chains of substrate proteins. SET- and MYND-domain containing protein 3 (SMYD3) is EZH2 target, and H3K4 methyltransferase plays a critical role in various human cancers by activating oncogenes and genes associated with cell-cycle regulation [158]. It has been shown that overexpression of SMYD3 protein correlates with an elevated level of transforming growth factor-*β*1, which is a key factor for reactivation of EBV [159]. Overexpression of SMYD3 was also associated with increased STAT3 activation in gastric cancer, suggesting a useful prognostic biomarker [86].

A substantial fraction of data demonstrates that EBV alters the expression of small class of 17-23 nucleotide noncoding endogenous RNA that functions in RNA silencing. Long primary microRNA (pri-miRNA) plays an important role in the miRNA synthesis through RNA polymerase II and III enzymes Drosh. In the nucleus, pri-miRNA ultimately generates the pre-miRNA intermediate and Dicer to form mature miRNA. Dysregulation of miRNAs at the posttranscriptional level is associated with perturbed cellular processes that lead to tumorigenesis. Upregulation or downregulation of several miRNAs including *piRNA-651*, *piRNA-823*, and *miRNA-222* has been demonstrated in gastric cancer [160]. Alteration of miRNA in gastric cancer tissue and blood samples is accompanied by disrupted cell cycle as well as enhanced cell proliferation and metastases, suggesting their critical role in tumorigenesis [160, 161]. It has been shown that during latent infection, EBV genome overexpresses pre-miRNAs known as miRNA-BamH1 fragment A rightward transcripts (BARTs), and such miRNAs confer a degree of resistance to proapoptotic drugs in patients undergoing radiation therapy and chemotherapy [162]. EBV *miRNA BART 18-5p* targets MAP3K2 and promotes viral persistence in vivo by suppressing viral replication in latently infected B-cells [161]. Finally, *miR-223* and *miRNA BART15* were found to activate the NLRP3 inflammasome and induction of IL-1*β* [163].

## 6. Summary

Further insight knowledge into the mechanisms linking the DNA damage, viral integration or propagation, and epigenetic alterations is required if potential targets for therapy are going to be developed. It is clear from the current literature that DNA damage facilitates viral-host integration especially with HPV and HBV viruses leading to genomic instability and perturbed cell cycle checkpoints that could contribute to uncontrolled cell growth and cancer development. ROS-induced activation of a pool of inflammasome cellular cascades, viral integration, and epigenetic regulators that allow viral manipulation of DNA repair machinery appears to be the common denominator in viral-induced carcinogenesis. Although EBV is not integrated into the host genome, its materials such as EBNA1 and BZLF1 seem to interact with various cellular promoters and induce epigenetic alterations that are associated with carcinogenesis. A combinatorial approach that will target the DNA damage and viral integration while reversing the epigenetic changes may be an effective treatment option for eradicating virus-based carcinogenesis.
